# Editorial: Epidemiology of diabetes in Asia

**DOI:** 10.3389/fcdhc.2025.1732167

**Published:** 2026-01-14

**Authors:** Cheow Peng Ooi, Norlaila Mustafa, Sueziani Binte Zainudin

**Affiliations:** 1 International Medical University, Kuala Lumpur, Malaysia; 2 Universiti Kebangsaan Malaysia, Bangi, Malaysia; 3 Sengkang General Hospital, Singapore, Singapore

**Keywords:** Asian phenotype, insulin resistance (METS-IR), metabolic biomarkers, multimorbidity, patient-centered care, resting heart rate (RHR), self-efficacy and health literacy, type 2 diabetes (T2D)

The global landscape of type 2 diabetes (T2D) is rapidly changing, with Asia emerging as a significant epicenter of the pandemic (1, 2). The narrative is familiar: rapid economic development, urbanization, and nutritional shifts have driven epidemics (3). The sheer number of patients with diabetes in this region, coupled with its unique phenotypic expression, early onset, lean individuals, complex multimorbidity patterns, and varied treatment responses, pose unprecedented challenges to healthcare systems and the economy (4–7). This Research Topic, “Epidemiology of Type 2 Diabetes in Asia,” explores this multifaceted issue through four distinct, yet interconnected studies, each offering valuable insights.

​One of the key findings from this Research Topic is the distinct metabolic phenotype prevalent in Asian populations. This phenotype typically includes younger age, lower body mass index (BMI), higher body fat percentage, elevated postprandial glucose levels, and poorer beta-cell function at onset (5, 8). Joblin-Mills et al. provided compelling molecular insights into the “Thin on the Outside, Fat on the Inside” (TOFI) phenomenon. This study examined postprandial metabolome responses to whey protein challenge in prediabetic Asian Chinese and Caucasian European women, moving beyond traditional static anthropometry, using untargeted metabolomic analysis and multivariate machine learning modeling. They found that ethnicity was a stronger driver of metabolic variance than intra-pancreatic fat deposition (IPFD). Chinese participants showed significant glycine depletion, independent of BMI, and dysregulated urea cycle metabolites (e.g., glutamic acid, ornithine, and arginine). These findings suggest impaired nitrogen handling and ammonia metabolism, which may increase vulnerability to T2D through insulin resistance, dyslipidemia, and ectopic fat accumulation influenced by genetics and other unknown factors (5). Although the study had limitations, such as a small sample size and female sex only, it highlights the unique susceptibility of Asian populations to T2D. These findings guide future research to explore the potential practical applications of glycine-supplemented diets or diets with reduced branched-chain amino acids to alleviate urea stress and hyperglycemia, postprandial glycine/uric acid monitoring for early risk assessment, and exercise to reduce ectopic fat and delay T2D onset.

Recognizing that the Asian phenotype may require earlier and more nuanced risk detection, pursuing simple, noninvasive, and scalable biomarkers is essential. Two pioneering studies in this Research Topic addressed these challenges. Chen et al. conducted an extensive retrospective cohort study that validated the Metabolic Score for Insulin Resistance (METS-IR) as a predictor of incident diabetes. Unlike current resource-intensive methodologies for measuring and estimating insulin resistance, the METS-IR index offers a cost-effective surrogate by combining routine measurements (fasting glucose, triglycerides, high-density lipoprotein cholesterol [HDL-C], and body mass index [BMI]) (9). Another study by Chen et al. established a significant association between higher resting heart rate (RHR) and inadequate glycemic control. This finding is particularly noteworthy because it suggests a bidirectional relationship between metabolic and autonomic health, which is often overlooked in routine diabetes care. However, whether RHR results from poor glycemic control or autonomic health remains unclear.

While both studies utilized large datasets, lending statistical power to bolster their conclusions, their cross-sectional (for RHR) and retrospective (for METS-IR) designs limit the ability to establish causation. While both studies utilized large datasets, lending statistical power to bolster their conclusions, their cross-sectional (for RHR) and retrospective (for METS-IR) designs limit the ability to establish causation. Nevertheless, the clinical implications of these cost-effective biomarkers warrant further investigation. Integrating RHR and METS-IR, examples of simple metrics, into electronic health records as automated flags could encourage earlier interventions. Thus, it challenges traditional resource-intensive screening models and suggests a path toward more efficient population-level risk assessments (9, 10).

In Asia, the T2D landscape is shaped by an aging population and the accelerated biological aging associated with the disease, bringing the challenge of multimorbidity into sharper focus (4, 11). Bing et al. conducted a cluster analysis involving elderly Chinese patients with T2D and multimorbidity, shifting the emphasis from risk prediction to the complexities of managing multiple conditions. By employing a patient-centered approach, the study moved beyond the HbA1c metrics and categorized participants into “good” (54.9%, n=189), “intermediate” (33.4%, n=115), and “poor” (11.6%, n=40) clusters based on self-reported depression, distress, self-efficacy, and health literacy. A striking paradox emerges: patients with higher self-reported depression and distress are often found among those categorized as “good” due to better control of their diabetes metrics, indicating the complex interplay between mental health and diabetes management. The self-perpetuating cycle plays a pivotal role in the amplification of psychological stress in patients with multimorbidity. This cycle arises when the emotional burden of managing multiple concurrent illnesses overwhelms an individual, thereby undermining their confidence in managing their health (i.e., self-efficacy). Emotional overload can lead to difficulties in adhering to prescribed treatment plans and ultimately impair glycemic control. Exacerbating factors include social isolation (e.g., living without a partner) and physical inactivity, heightening overall vulnerability. The “poor” cluster exemplifies this, with peak depression and distress, diminished self-rated health and self-efficacy, driven by predictors such as advanced age, male sex, cerebral infarction, and lower BMI, illuminating the ruinous synergy between macrovascular complications and mental health. Although frequent healthcare engagement enhances knowledge, it often neglects the profound emotional strain of navigating multifaceted diseases. These insights dismantle the one-size-fits-all diabetes management approach, advocating a stratified framework for developing tailored interventions for vulnerable T2D patients. The findings highlight the pressing need for a more integrated approach to T2D care in Asia, emphasizing the importance of addressing physical and mental health components to improve overall patient outcomes.

​​In conclusion, the four articles collectively articulate a coherent narrative: the T2D epidemic in Asia is characterized by unique pathophysiological features and requires a multifaceted and region-specific approach. Evidence converges on several critical points: a distinct metabolic phenotype predisposes the Asian population to T2D, simple and accessible biomarkers hold significant potential for enhancing risk stratification, and the increasing burden of multimorbidity necessitates a transition toward person-centered care models. Although not exhaustive, this body of work critically frames the current challenges. This curated Research Topic provides a catalyst that will inspire key stakeholders in diabetes care to engage in critical reflections and collaborative discussions, ultimately advancing innovative studies on the unique epidemiological patterns of diabetes across diverse ethnic groups in Asia.
